# Structural Iron (II) of Basaltic Glass as an Energy Source for Zetaproteobacteria in an Abyssal Plain Environment, Off the Mid Atlantic Ridge

**DOI:** 10.3389/fmicb.2015.01518

**Published:** 2016-01-21

**Authors:** Pauline A. Henri, Céline Rommevaux-Jestin, Françoise Lesongeur, Adam Mumford, David Emerson, Anne Godfroy, Bénédicte Ménez

**Affiliations:** ^1^Institut de Physique du Globe de Paris, Sorbonne Paris Cité, Univ Paris Diderot, Centre National de la Recherche ScientifiqueParis, France; ^2^Laboratoire de Microbiologie des Environnements Extrêmes, Ifremer, CNRS/UMR 6197Plouzané, France; ^3^Bigelow Laboratory for Ocean SciencesEast Boothbay, ME, USA

**Keywords:** basaltic glass, Zetaproteobacteria, bio-mediated alteration, iron-oxidation, abyssal plain

## Abstract

To explore the capability of basaltic glass to support the growth of chemosynthetic microorganisms, complementary *in situ* and *in vitro* colonization experiments were performed. Microbial colonizers containing synthetic tholeitic basaltic glasses, either enriched in reduced or oxidized iron, were deployed off-axis from the Mid Atlantic Ridge on surface sediments of the abyssal plain (35°N; 29°W). *In situ* microbial colonization was assessed by sequencing of the 16S rRNA gene and basaltic glass alteration was characterized using Scanning Electron Microscopy, micro-X-ray Absorption Near Edge Structure at the Fe-K-edge and Raman microspectroscopy. The colonized surface of the reduced basaltic glass was covered by a rind of alteration made of iron-oxides trapped in a palagonite-like structure with thicknesses up to 150 μm. The relative abundance of the associated microbial community was dominated (39% of all reads) by a single operational taxonomic unit (OTU) that shared 92% identity with the iron-oxidizer *Mariprofundus ferrooxydans* PV-1. Conversely, the oxidized basaltic glass showed the absence of iron-oxides enriched surface deposits and correspondingly there was a lack of known iron-oxidizing bacteria in the inventoried diversity. *In vitro*, a similar reduced basaltic glass was incubated in artificial seawater with a pure culture of the iron-oxidizing *M. ferrooxydans* DIS-1 for 2 weeks, without any additional nutrients or minerals. Confocal Laser Scanning Microscopy revealed that the glass surface was covered by twisted stalks characteristic of this iron-oxidizing Zetaproteobacteria. This result supported findings of the *in situ* experiments indicating that the Fe(II) present in the basalt was the energy source for the growth of representatives of Zetaproteobacteria in both the abyssal plain and the *in vitro* experiment. In accordance, the surface alteration rind observed on the reduced basaltic glass incubated *in situ* could at least partly result from their activity.

## Introduction

In the dark ocean, microbial communities must thrive either by using organic carbon coming from the photic zone as an energy source, thus by chemoorganotrophy or by chemolithotrophy. Chemolithotrophic microbes gain their energy by oxidizing a wide variety of reduced inorganic substrates such as sulfur and sulfides, ammonia, molecular hydrogen, and iron. Because iron is the fourth most abundant element in the Earth’s crust, Fe(II) is potentially among the most abundant energy sources for life at the boundary between the oxygenated ocean and the reducing subsurface (Fortin and Langley, 2005). Nonetheless, the chemical oxidation of Fe(II) to less soluble Fe(III) and the subsequent precipitation of iron (oxyhydr)oxides occur rapidly in the presence of strong oxidants especially at non-acidic pH in well-oxygenated environments. The iron oxidizing bacteria (FeOB) that derive their energy from the oxidation of Fe(II) compete with this abiotic reaction; however, when the O_2_ fugacity is low, specialized groups of FeOB can flourish.

Microbial Fe(II)-oxidation has a lower energetic yield (≈-90 kJ⋅mol^-1^; Emerson et al., 2010) compared to other chemolithotrophic metabolisms that are widespread at the ocean/seafloor interface such as sulfide oxidation (≈-500 kJ⋅mol^-1^; Edwards et al., 2005). Despite these poor energetics and a well oxygenated modern ocean, communities of marine FeOB are increasingly being described, especially in areas of hydrothermal venting, either at seamounts or at seafloor spreading centers, where anoxic and hot fluids enriched in Fe(II) discharge and mix with oxygenated cold seawater (Emerson and Moyer, 2002; Hodges and Olson, 2009; Kato et al., 2009; Sudek et al., 2009; Emerson et al., 2010). Among these FeOB, members of the newly described class formed by the Zetaproteobacteria (Emerson et al., 2007) were shown to be widely distributed and systematically associated with iron-rich marine environments (Scott et al., 2015). Zetaproteobacteria are able to grow via Fe-oxidation at circumneutral pH and under microaerophilic conditions, resulting in the precipitation of large amounts of Fe-oxyhydroxides. One cultured representative of this class, *Mariprofundus ferrooxydans* PV-1, produces characteristic twisted stalks of iron oxyhydroxides that also contain exopolymeric substances (Emerson and Moyer, 1997; Chan et al., 2009).

While the biological oxidation of Fe(II) is now well established in certain deep-sea benthic habitats, there are still open questions about the sources of Fe(II). Edwards et al. (2003) were the first to test the hypothesis that glassy basalt could support the growth of FeOB, acting as a substrate by constituting a solid Fe(II) source. They carried out *in vitro* experiments during which they isolated FeOB from deep sea, low-temperature weathering deposits recovered in the vicinity of the Juan de Fuca hydrothermal area. These bacteria were cultured on a variety of natural and synthetic solid rock and mineral substrates, including basaltic glass (at 10 wt% FeO) without any addition of other energy sources. It was shown that when the basaltic glass was used as substrate, it promoted the growth of neutrophilic autotrophic iron-oxidizing Gammaproteobacteria- and Alphaproteobacteria, suggesting a role for these organisms in primary production and basalt weathering at seafloor. However, neither the initial basalt substrate nor the obtained alteration by-products were characterized or discussed. In parallel, several studies that aimed at describing microbiota hosted in basalts, demonstrated that microbial communities of the oceanic crust are very diverse (Lysnes et al., 2004; Templeton et al., 2005; Mason et al., 2007, 2009; Einen et al., 2008; Santelli et al., 2008; Sudek et al., 2009; Orcutt et al., 2011). However, there have few attempts to link the inventory of the microbial communities with the description of the basalt alterations phases. It was shown that the microbial diversity positively correlates with the degree of basaltic glasses’ alteration (Santelli et al., 2008), and that the composition of the microbial communities depended on the age of the basalts and therefore on their alteration state (Lysnes et al., 2004; Mason et al., 2009). Nonetheless these studies mainly focused on old basalts (of millions of years) recovered by drilling into the oceanic crust.

Few studies focused on young oceanic basaltic glasses. Those have shown that, although abundantly colonized by heterotrophic microorganisms, the glassy rims of the basaltic lavas were not significantly altered (Templeton et al., 2009; Cockell et al., 2010). Consequently, the basaltic glass was considered by these authors to be a surface habitat on which to grow, rather than as a direct source of energy to support microbial growth. In parallel, *in situ* and laboratory experiments have also demonstrated the capability of adhering microorganisms to directly alter silicates and hence to release elements from the basalt by producing protons, hydroxyl groups or organic ligands at the point of attachment. By modifying locally their environment through their metabolic by-products, microorganisms can then make bioavailable the nutrients and metals contained in silicates (Bennett et al., 2001; Brantley et al., 2001; Rogers and Bennett, 2004).

Here, we present the first direct evidence of the capability of structural Fe(II) from basaltic glass to serve as an energy source for FeOB belonging to the class of Zetaproteobacteria in an abyssal plain off the Mid Atlantic Ridge (MAR). In this context, bottom seawater contrasts with seafloor environments influenced by hydrothermal fluids, being typified by low temperatures, high chlorinity, high pH, high alkalinity, and low metal and hydrogen sulfide concentrations. The results reported here combine complementary *in situ* and *in vitro* colonization experiments whose products were characterized by a multidisciplinary approach that combined molecular ecology along with spectroscopy and microscopy techniques.

## Materials and Methods

### Experimental Settings

#### *In Situ* Experiment

**FIGURE 1:**
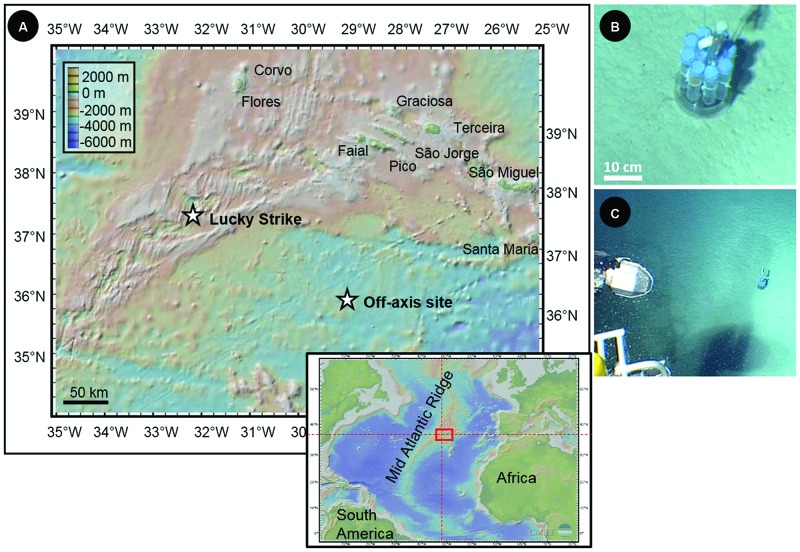
**(A)** Location and associated bathymetry of the Lucky Strike hydrothermal field along the Mid-Atlantic Ridge (MAR) and of the off-axis site in the Atlantic abyssal plain (N35°59, 88; W29°02,949; 3200 mbsl) where incubations of synthetic basaltic glasses were carried out during 11 months. **(B)** and **(C)** Pictures of the microbial incubator deployed in the abyssal plain (taken in 2006 by the *Nautile* submersible).

The *in situ* colonization experiment was carried out for 11 months in a sedimented abyssal plain, off the Mid Atlantic Ridge (N35°59,88; W29°02,949 at 3200 m below sea level – mbsl; **Figure 1**). The deployment of the colonization device was done during the Graviluck 06 cruise (R.V. *L’Atalante* with the *Nautile* submersible, 2006) and the recovery occurred during the MoMARDream 07 cruise (R.V. *Pourquoi Pas?* with the *Nautile* submersible, 2007). During the Graviluck 06 cruise, a CTD profile was collected up to 2635 mbsl in the water column above the deployment location, in order to characterize the environmental conditions of the site.

The colonization modules consisted of a ballasted plastic holder that hosted up to twelve biological colonizers and abiotic controls distributed around an autonomous temperature probe. The biological incubators were made up of capped 50 ml DB Falcon^TM^ conical polypropylene tubes, in which ±20 holes of 1 mm in diameter were drilled to allow seawater circulation. Each colonizer was filled with chips of basaltic glass designed to serve as solid Fe-substrates. The basaltic glasses were synthesized at the Laboratoire de Géomatériaux et Environnement (Université de Marne-la-Vallée, France) from a mixture of different oxide and carbonate powders, resulting in a typical tholeiitic basalt composition (i.e., in weight%, 48.6 SiO_2_, 15.7 Al_2_O_3_, 11.1 CaO, 7.7 MgO, 12.5 FeO/Fe_2_O_3_, 2.7 Na_2_O, 0.2 K_2_O, and 1.4 TiO_2_). Two different types of synthesis were performed: (1) under a reducing atmosphere of H_2_ to enrich the glass in Fe(II) (hereafter referred to as BH2) and (2) under an oxidizing atmosphere of O_2_ to conversely enrich the glass in Fe(III) (hereafter referred to as BO2). The synthesis protocol is described in the Supplementary Material. Freshly synthetized basaltic glasses were cleaned in an ultrasonic bath filled with ethanol, and then air-dried before being packed in the colonizers. In order to benefit from abiotic controls, tubes without holes were also filled with basaltic glass chip and closed by a 0.22 μm cellulose filtration membrane (Millipore^TM^) to allow exchanges with the surrounding fluid while avoiding microbial colonization. The filled colonizers were sterilized by autoclaving for 30 min at 121°C shipboard. The colonizer holder was cleaned up with a Desibac HPC^®^ solution and rinsed with deionized water (MilliQ^TM^ 18 MΩ) then ethanol 96% prior to the assemblage. For the deployment by the Nautile submersible during the Graviluck cruise (2006), the full module was installed in a biobox that was cleaned with the same protocol as for the colonizer holder and filled with sterilized deionized water in order to avoid microbial contamination from the water column during operations. During the incubation period, *in situ* temperature was monitored every 5 min by using an autonomous temperature sensor (NKE SDT6000D-26001) set amongst the Falcon^TM^ tubes.

During the MoMARDream cruise (2007), the module was recovered in a biobox using the Nautile submersible and the incubated basaltic glass chips were preserved on board under sterile conditions for different types of analysis. For DNA analysis, samples were stored in ethanol 96% at -20°C. About 2 L of ambient seawater from the seafloor was filtered onto 0.22 μm-pore-size membranes, which were then stored in ethanol 96% at -20°C for DNA analyses. Deep sea sediments were also sampled in the vicinity of the colonization module and similarly preserved for DNA analysis.

#### *In Vitro* Experiments

Two types of *in vitro* experiments were conducted. The first one aimed at reproducing in laboratory the conditions mimicking the abiotic control which did not succeed during the *in situ* experiment. For this purpose, fragments of reduced basaltic glass were autoclaved 20 min at 121°C and packed in 50 ml Falcon^TM^ tubes containing sterile costal seawater (previously filtered onto 0.22 μm filtration membrane and autoclaved). One tube was incubated at 4°C whereas a second one stayed at ambient temperature, both during 1 year.

The second experiment aimed at testing the hypothesis that Zetaproteobacteria could thrive with the synthetic reduced basaltic glass as the only solid Fe(II) substrate. For this purpose a custom built flow through system was used. Briefly, this consisted of a 50 ml incubation chamber that was continuously flushed with sterile seawater, buffered with NaHCO_3_ at pH 6.5 and gently bubbled with filtered air to avoid complete anaerobic conditions (Mumford and Emerson, unpublished results). Two sterilized chips of synthetic reduced basaltic glass were stuck on a glass slide with silicon glue. The slide was introduced vertically in the incubation chamber that was inoculated with 1 ml of a pure culture of the FeOB *M. ferrooxydans* DIS-1 that was originally isolated from mild steel, and is an obligate Fe-oxidizer. Prior to inoculation, abundance and shape of the cells were checked through fluorescent staining using the green-fluorescent nucleic acid stain SYTO^®^13. The incubation lasted 2 weeks, without any addition of nutrient, chemical or minerals.

### Methods and Data Analyses

#### Analysis of the 16s rRNA Gene Sequences

In order to characterize the microbial diversity, total genomic DNA was extracted using the UltraClean^®^ Soil DNA Isolation Kit (MO BIO laboratories, Inc,) following the manufacturer protocol.

Sequencing of the 16S rRNA gene sequences of the community DNA extracted from the reduced and oxidized incubated basaltic glasses (BH2 and BO2, respectively), the abyssal plain sediment (SED) and the background seawater samples (H2O) was performed by conventional Sanger technique using universal primers: U1492R (5′-GGC TAC CTT GTT ACG ACT T-3′) as reverse primer and E8F (5′-AGA GTT TGA TCC TGG CTC AG-3′) or 27F (5′-AGA GTT TGA TCC TGG CTC AG-3′) as forward primers. Clone libraries were constructed using the TOPO^®^ XL PCR Cloning Kit, with the One Shot^®^ TOP10 Chemically Competent *Escherichia coli* kit (Invitrogen^TM^) according to the manufacturer instructions. Plasmid extraction, purification and sequencing were carried out by GATC Biotech (Germany).

The microbial diversity of BH2 sample was additionally analyzed by 454-pyrosequencing through the amplification of the V2–V3 region of the 16S rRNA coding genes with 27F (5′-AGA GTT TGA TCC TGG CTC AG-3′) as forward primer and 533R (5′-TTA CCG CGG CTG CTG GCA C-3′) reverse. Pyrosequencing, demultiplexing, and contig assembly were carried out by Beckman Coulter genomics (Danvers, MA, USA) using the Roche GS FLX platform (454 Life Sciences, Branford, CT, USA) with the Titanium LIB-A kit for bi-directional amplicons sequencing (see Supplementary Material for further information).

Data processing was performed with Mothur (Schloss et al., 2009). For 454-pyrosequencing analyses, we only kept the sequences whose sizes ranged between 400 and 500 bp with no ambiguity and a maximum homopolymer length of 8 bp. A 50 bp sliding-window with an average quality of 35 was used for filtering, as recommended in (Schloss et al., 2011). For all sequences, the presence of chimeras for removal was checked by Uchime (Edgar et al., 2011). Taxonomic affiliations were made with the SILVA database (Pruesse et al., 2007; Quast et al., 2012). Sequences with bootstrap values below 95% have been considered as non-affiliated sequences. Sequence alignment, generation of the distance matrix from the aligned sequences and calculation of the rarefaction curves and richness indicators were performed with Mothur (v1.33.3). The sequence data reported in this study have been submitted to the GenBank nucleotide sequence database under accession number KM580076–KM580346 for the sequences obtained by Sanger sequencing and the BioProject ID PRJNA260775 for the sequences obtained by 454-pyrosequencing.

A distance level of 0.03 was used to compare sequences for both rarefaction and diversity indicators. Operational taxonomic units (OTUs) were similarly defined by a 0.03 distance level (i.e., sequences with ≥97% similarity are designated to a single OTU). Representative sequences of each sample along with closely related environmental clones and cultured species were selected for general phylogenetic tree construction. Tree topology and branch lengths were determined with MEGA 5 (Kumar et al., 2008), using maximum likelihood criterion for distance analysis. Maximum likelihood bootstrapping was carried out with 1,000 replicates.

#### Spectroscopy/Microscopy

Raman spectroscopy analyses were performed at IPGP with a Renishaw InVia spectrometer using the 514 nm wavelength argon laser (20 mW) focused through an Olympus BX61 microscope with 50× objective (numerical aperture: 0.75). The obtained planar resolution was about 1 μm, with a power delivered at the sample surface of 0.5 mW. The Raman signal was dispersed with a 1800 grooves/mm holographic grating and collected using a RENCAM CCD array detector. The obtained spectral data were acquired, visualized and the baselines were subtracted (by polynomial fitting) with the software Wire 3.3 (Renishaw).

Scanning Electron Microscopy (SEM) was performed at the “Service Commun de Microscopie Electronique à Balayage” (UPMC, Paris, France) with a Zeiss SUPRA 55 VP Field Emission Scanning Electron Microscope on carbon coated samples. Three secondary electron detectors (Everhart-Thornley for high voltage mode, VPSE for variable pressure mode and InLens for low voltage mode) and a backscattered electron detector enabled the acquisition of high resolution images using analytical conditions that varied from 3 to 30 kV, 10 pA–1 nA, and 30–133 Pa with a 3.3–7.2 mm working distance. Elemental microanalyses were also performed using an Energy Dispersive X-ray (EDX) spectrometer (PGT Sahara).

To assess the redox state of iron in the synthetic basaltic glasses before and after the *in situ* incubation experiment, glass chips were embedded in LR White resin (4/5 araldite DBF epoxy resin, 1/5 hardener Huntsman containing triethylenetetramine) following the manufacturer protocol. Embedded chips were then cut and polished first with a silicon carbide polishing paper and then on tissue carpet with a solution of Mecaprex^®^ diamond compounds up to a 1 μm grain until glass chips were outcropping. Synchrotron-based X-ray fluorescence measurements of elemental distributions and X-ray Absorption Near Edge Structure (XANES) spectra at the Fe K-edge were acquired on beamline LUCIA (Synchrotron Soleil, Saint Aubin, France) using Si (311) crystal monochromator (0.2 eV resolution for Fe) and a focused spot size of 2 μm × 2 μm. Several Fe-bearing compounds with various redox states were used as references (see Supplementary Table S1). The iron redox state of samples was evaluated following the procedure described by Wilke et al. (2001; see Supplementary Figure S2 and Table S2). Spectra collected on the alteration rind were fitted using linear combinations of the Fe K-edge XANES spectra collected on the reference compounds in order to determine the composition of the alteration layer.

Confocal Laser Scanning Microscopy (CLSM) was used to visualize basalt chip surfaces from the *in vitro* incubation experiments. Each sample was overlaid with a staining solution consisting of 1.5 μl at 2 mg/ml of rhodamine-conjugated *Ricinus communis* agglutinin I (Vector Labs), and 5 μl of SYTO^®^13 in 144.5 μl 0.5% low-melting-point agarose in MilliQ reagent-grade water. They, respectively, stain carbohydrate linkages in polysaccharides, and nucleic acids. Following the application of the staining solution, a custom-made frame allowed for approximately 150 μm of clearance between the top of the basaltic glass chip and the underside of the coverslip (Mumford and Emerson, unpublished results). After the frames were applied, the slides were incubated with the dyes for 1 hour at 4°C, and then low melt agarose (0.5%) was added to stabilize the chips and covered with a glass coverslip. Observations were performed at Bigelow Laboratory with a Zeiss LSM 700/Axio Observer using an oil immersion C-Apochromat objective 40×/1.2 W. Fluorescence images were obtained following excitation at 488 and 555 nm, by collecting the emitted fluorescence between 300–550 and 578–800 nm, respectively.

Additional CLSM images were acquired at IPGP using an Olympus FluoView FV1000 Confocal Microscope, displaying a spectral resolution of 2 nm and a spatial resolution of 0.2 μm, on samples from the *in situ* experiment stained with green-fluorescent SYTO^®^9 used at a working concentration of 50 μM. An oil immersion objective UPLSAPO 60XO (Olympus; 60× magnification, numerical aperture = 1.35) was used. Fluorescence image stacks were obtained with excitation at a wavelength of 488 nm, by collecting the emitted fluorescence between 300 and 500 nm. The three-dimensional images were acquired, visualized and processed using the F10-ASW FLUOVIEW software (Olympus).

## Results

The colonization module was deployed in the abyssal plain on carbonated sediments (**Figures 1B,C**). The oxygen concentration measured by the CTD probe at 2635 mbsl was 246.8 μmol/kg, attesting to a well-oxygenated environment. The temperature recorded by the probe installed in the colonization module revealed stable values during the entire incubation period (i.e., 11 months) with a mean value of 2.92°C (±0.02°C).

### Phylogenetic Diversity and Distribution for the *In Situ* Experiment

For BH2, the incubated synthetic reduced basaltic glass for which both sequencing techniques were used, 454-pyrosequencing provided as expected more reads (3797) compared to the number of clones retrieved by Sanger sequencing (42). Because of this greater number of sequences, allowing a more representative assessment of the bacterial diversity and composition for BH2, we have focused on the 454-pyrosequencing results in the case of this sample. For comparison with the diversity results obtained for the three other samples that were only sequenced using the Sanger technique, we have only used percentages of the total number of the obtained sequences for each sample. Both sequencing techniques lead to comparable OTU distribution for BH2 (**Table 1**). Shannon indices were also comparable for the two sequencing techniques (**Table 2**).

**Table 1:** Distribution of phyla and classes retrieved from the oxidized and the reduced basaltic glass incubated *in situ* (BO2 and BH2), the sediment (SED) and the water (H2O) samples using Sanger sequencing (Sang) and pyrosequencing (Pyr).

Phylum^1^	Class^1^	Number of OTUs					Percentage of total sequences				
		BH2		BO2	H2O	SED	BH2		BO2	H2O	SED
		Pyr	Sang	Sang	Sang	Sang	Pyr	Sang	Sang	Sang	Sang
Proteobacteria	α	90	10	6	12	7	18	36	32	80	13
	γ	88	3	3	3	13	6	12	10	3	23
	ζ	1	1				39	31			
	δ	11		3	1	4	0.5		6	1	6
	𝜀	30	2				7	7			
	β	10			1	2	0.4			1	3
	JTB23				1					1	
	Others	34			1	2	1			1	3
Bacteroidetes		111	4	8		1	21	12	23		2
Planctomycetes		37	1	3		9	2	2	11		14
Acidobacteria						2					3
Actinobacteria		3		1	3	1	0.2		2	7	2
Candidate_division_TG-1						1					2
Chloroflexi		2				3	0.1				6
Gemmatimonadetes						1					5
Nitrospirae						2					3
Verrucomicrobia		3		1			0.1		2		
Other classes^2^		12					0.4				
Non affiliated after the domain		63		3	2	9	3		12	5	16

*α, γ, ζ, δ, ε, β stand for Alphaproteobacteria, Gammaproteobacteria, Zetaproteobacteria, Deltaproteobacteria, Epsilonproteobacteria, and Betaproteobacteria, respectively*.

*^1^Representing more than 1% of the total sequences at least in one sample*.

*^2^Representing less than 1% of the total sequences in the samples*.

**Table 2:** Number of OTUs retrieved in the oxidized and the reduced basaltic glass incubated *in situ*, the sediment and the water samples using Sanger sequencing and pyrosequencing (pyro) along with estimates of diversity and richness.

	Collected sequences	OTU observed	Shannon H′ (CI)	Chao1 (95% CI)
Water (Sanger)	87	24	2.51 (±0.25)	70 (36–195)
BO2 (Sanger)	81	28	3.11 (±0.18)	36 (31–52)
BH2 (Sanger)	42	21	2.77 (±0.33)	38 (27–74)
BH2 (pyro)	3797	495	3.23 (±0.08)	1161 (981–1408)
Sediment (Sanger)	64	57	4.00 (±0.18)	270 (145–570)

*CI stands for confidence indices*.

**FIGURE 2:**
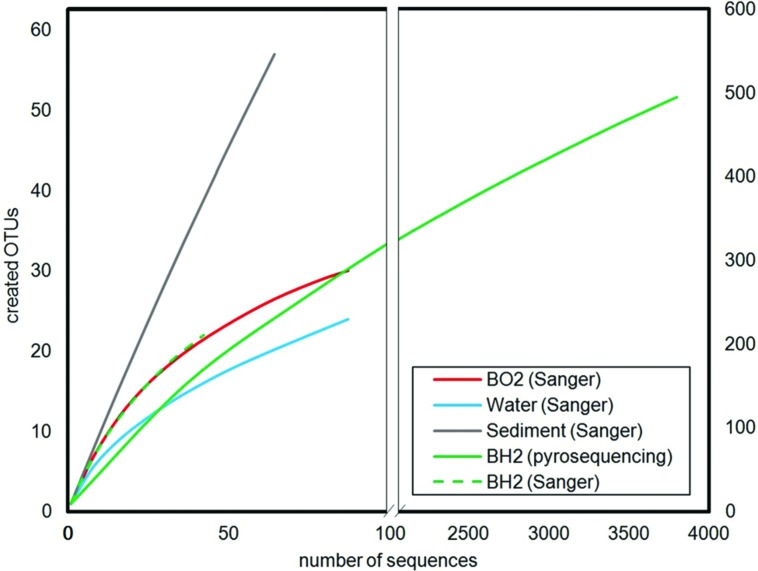
**Rarefaction curves calculated for a 0.03 distance level**. The left part corresponds to an enlarged view of the number of OTUs created for a number of analyzed sequences below 100 in order to allow the comparison between the low number of sequences obtained by Sanger sequencing for seawater, sediment and basaltic glasses, and the high number of sequences obtained by 454-pyrosequencing for the reduced basaltic glass.

The Shannon’s indices estimated for each sample showed that the microbial diversity in the water sample was the lowest. Those of the two incubated basaltic glass were comparable with intermediate values, the greatest values being that of the sediment (**Table 2**). By considering only the Sanger results, the observed and predicted OTU richness also showed more diversity in the sediment, followed by seawater and then the two basaltic glass samples. The rarefaction curves depicting the number of OTUs as a function of the number of analyzed sequences (**Figure 2**), showed that for an equivalent sampling effort (<100 sequences, left part of the plot) the highest observed species richness was found in the sediment, its rarefaction curve being far from reaching an asymptote, while those of BH2 (for both Sanger and 454-pyrosequencing), BO2 and water were comparable.

For all samples, most of the sequences were not closely related to cultured microorganisms. The high level (phylum/class) taxonomic distribution of OTUs is shown in **Table 1**. The phylum *Proteobacteria* dominated in all the samples and represented 48% of the OTUs retrieved from sediment and BO2 and 72–87% of the OTUs of BH2 and water, respectively. The only classes represented in all the samples were Alphaproteobacteria, Deltaproteobacteria, Gammaproteobacteria, and *Actinobacteria*. The classes with the highest relative abundance were Alphaproteobacteria in water (80%) and BO2 (32%), Gammaproteobacteria in the sediment (23%) and Zetaproteobacteria for BH2 (39%). The incubated basaltic glasses BH2 and BO2 accounted for 21 and 23% of the retrieved *Bacteroidetes* sequences, respectively, while this phylum represented only 2% of the retrieved sequences in the sediment and was absent in water.

#### Basaltic Glass Samples

**FIGURE 3:**
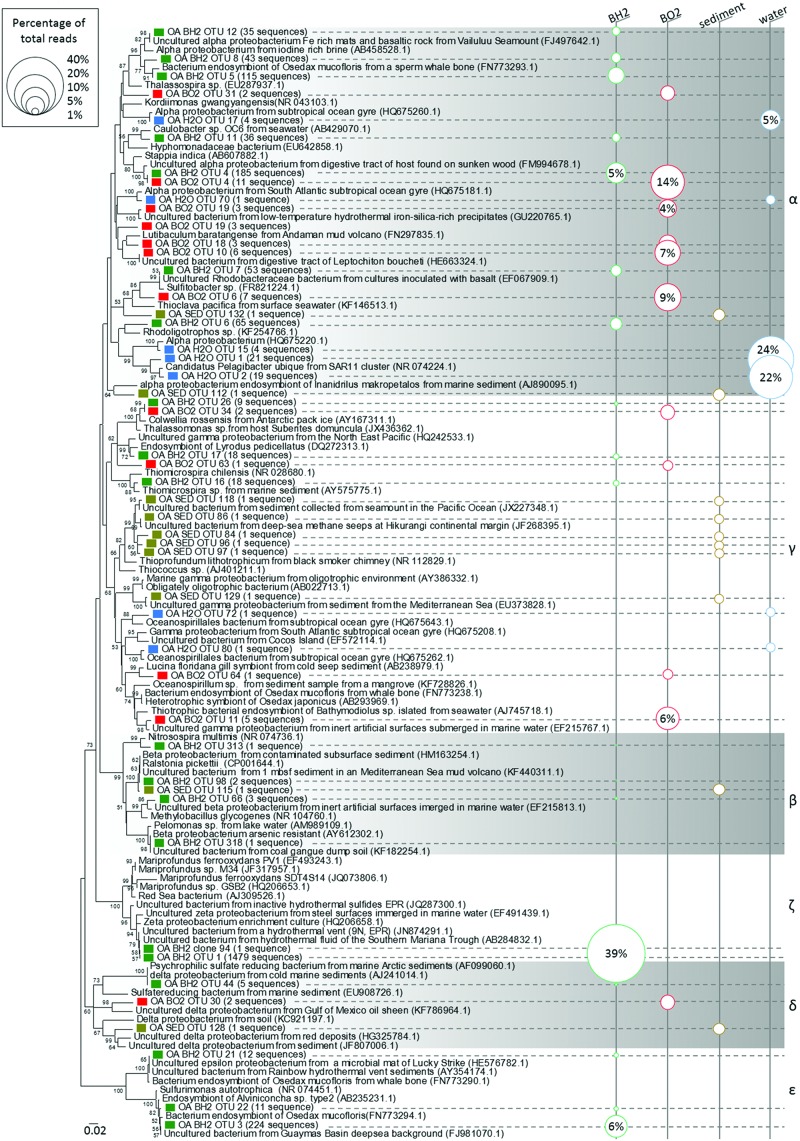
**Phylogenetic relationships among the proteobacterial 16S rRNA gene sequences of representative OTUs of the oxidized (BO2) and reduced (BH2) basaltic glass incubated in the abyssal plain along with the seawater (H2O) and the sediment (SED).** Sequences obtained in this study and designated by a prefix “OA” are indicated by a colored square; Green = BH2; Red = BO2; Blue = H2O; Bright brown = SED. The corresponding number of sequences obtained for each OTU is indicated after the name of the representative and the size of the circles in the right part figures the percentage represented by each OTU with respect to the total number of sequences. The numbers in the parentheses are the GenBank accession numbers for the NCBI sequences. The tree was determined by neighbor-joining analysis. Bootstrap values for nodes were obtained using 1,000 replicates, and only values >50% are indicated. The scale bar represents 0.02 substitutions per nucleotide position. α, γ, β, ζ, δ, and ε stand for Alphaproteobacteria, Gammaproteobacteria, Betaproteobacteria, Zetaproteobacteria, Deltaproteobacteria, and Epsilonproteobacteria, respectively.

Zetaproteobacteria were only retrieved in the reduced iron basalt sample (BH2). A single OTU accounted for 39% of the total sequences retrieved from this sample, while the next most abundant OTU accounted for 21% of the sequences (**Table 1**). The 400 bp read for the Zetaproteobacteria OTU (sequence OA BH2 OTU 1 in **Figure 3**) obtained by 454-pyrosequencing was nearly identical to almost complete 16S rRNA gene sequence obtained by Sanger sequencing (OA BH2 clone 94 in **Figure 3**). It was 99% similar to a sequence identified as Zetaproteobacteria sampled from a hydrothermal fluid near the Southern Mariana trough (AB284832.1, **Figure 3**; Kato et al., 2009). The nearest cultured organism (with 92% similarity) was *M. ferrooxydans* PV-1, known to be a chemolithoautotrophic iron oxidizing bacterium (Emerson and Moyer, 1997).

Members of the Epsilonproteobacteria, also only present in BH2 sample (**Figure 3**), were taxonomically affiliated to the sulfide-oxidizing genera *Sulfurimonas* and *Sulfurovorum*. Sequences were related at 94 to 99% to endosymbionts of either *Osedax mucofloris* (FN773294.1) or *Alvinichonca* sp. from sunken woods (AB235231.1) or at 99% to uncultured bacteria from the Guaymas basin (FJ981070) and the Rainbow and Lucky Strike hydrothermal fields (AY354174.1 and HE576782.1, respectively).

As shown in **Figure 3** and **Table 1**, Alphaproteobacteria showed the highest relative abundance in BO2 sample (32%) and represented 18% of the total sequences retrieved from BH2. In BO2 the majority of alphaproteobacterial sequences (22%) were closely related to uncultured bacteria hosted in macrofauna found on sunken wood (FM994678.1; HE663324.1). The other alphaproteobacterial sequences were closely related to sequences collected either in deep-sea sediment or in low-temperature hydrothermal precipitates (FN297835.1; EF067909.1). Six percent of the alphaproteobacterial sequences in BH2 sample were closely related to uncultured bacteria hosted by gastropods also found on sunken woods (FM994678.1), and four percent to a chemoorganotrophic endosymbiont of the bone-eater worm *O. mucofloris* (FN773293.1). Some sequences were related to *Caulobacter* sp. that are ubiquitous in seawater and mainly chemoorganotrophic aerobes, or to a *Rhodobacteraceae* (EF067909.1) from aerobic enrichment cultures inoculated with basalt. A few other sequences belonged to the microaerophilic *Rhodospirillales* order, known to be photoorganotrophic but able to growth also without light. Among these sequences, some were closely related to an uncultured bacterium from Fe-rich microbial mats and basaltic rocks from Vailulu’u seamount (FJ497642.1).

Within the *Bacteroidetes* whose cultured representatives are heterotrophs and mostly aerobes, most of the BH2 sequences belonged to the *Flavobacteria* class, whereas in BO2, *Sphingobacteria* were more frequently retrieved than *Flavobacteria*. In BH2, most of the flavobacterial sequences were related to sequences found associated with macrofauna (Supplementary Figure S1). In BO2, some of these sequences were related to uncultured *Bacteroidetes* from chimney-like structures with iron oxides (FJ905648.1) or hydrothermal vents of the Lau basin (AB247861.1). Other sequences were closely related to the strictly aerobic chemoheterotroph *Gaetbulibacter marinus* (AB681678.1) and to an uncultured bacterium from deep sea vent (AY373402.1).

In the basalts BO2 and BH2, the gammaproteobacterial sequences (10 and 6% of the total sequences, respectively; **Table 1**) were mainly affiliated to the genera *Colwellia* (aerobic chemoorganotroph; AY167311.1) and to sulfur-oxidizing *Leucothrix* (**Figure 3**). Other sequences were related to the sulfur-oxidizing symbiont of the clam *Lucina florida.* In BO2, the aerobic gender *Oleiphilus*, thriving only on hydrocarbons and derivates (Golyshin et al., 2002), was also represented with sequences close to an uncultured *Oleiphilus* sp. from surface water samples of an iron fertilization experiment.

The phylum *Planctomycetes* represented, respectively, 11–14% of BO2 and sediment diversity and only 2% for BH2 (**Table 1**). The cultured representatives of this phylum include aerobic or facultative anaerobic chemoheterotrophs. As shown in Supplementary Figure S1, BO2 sequences of *Planctomycetes* were mainly related to uncultured bacteria from low temperature hydrothermal oxides of the South West Indian Ridge (JN860365.1), or to a lower extent to uncultured bacteria hosted by the marine sponge *Haliclona* cf. *Gellius* sp. (EU236275.1).

Deltaproteobacteria (**Figure 3**) in BO2 (6%; **Table 1**) were related to genera *Haliangium* (cultured species are myxobacteria), *Kofleria* and *Bdellovibrio* sp (parasitic). The deltaproteobacterial sequences represented only less than 1% of the diversity of BH2 and water, respectively.

The actinobacterial sequences in BO2 (2.5%) are close to an uncultured bacterium from arctic surface sediment (Supplementary Figure S1). They were absent from BH2 sample.

#### Sediment and Water Samples

Gammaproteobacteria were dominant in the sediment (23%) whereas they represented less than 10% of the other samples’ abundance. Numerous sediment gammaproteobacterial sequences were related to sequences retrieved from deep-sea polymetallic nodules and adjacent sediments (Wu et al., 2013). In the water sample, the 3% of Gammaproteobacteria were found either non-affiliated at the class level or as belonging to the oligotrophic marine cluster OM182.

The strong dominance of Alphaproteobacteria in the water sample was due to the affiliation of about 72% of the retrieved sequences to the heterotrophic SAR11 clade, widespread in oceanic environment and thriving on dissolved organic carbon and nitrogen from the water column (Morris et al., 2002). The water sample sequences were close to the SAR11 clade member Candidatus *Pelagibacter* (NR 074224.1, **Figure 3**). In the sediment alphaproteobacterial sequences (13%) belonged mostly to the families *Rhodobacteraceae* and *Rhodospirillaceae* and are closely related to sequences retrieved in deep sea polymetallic nodules or sediments.

The *Planctomycetes* sequences retrieved from the sediment (14%) are closely related to uncultured bacteria collected in marine sediment or abyssal plain water. *Planctomycetes* were absent in the water sample. The deltaproteobacterial sequences retrieved from the sediment (6%) are related at 84 to 90% to cultured iron-reducing *Geobacter* sp. or *Desulfuromonas* sp. reducing elemental sulfur into sulfide. The *Bacteroidetes*, well represented in the basaltic glasses, represent only 2% of the sediment’s OTUs and is absent in water. *Actinobacteria* were mainly represented in water (7%) with sequences affiliated to the *Acidimicrobineae* marine group and closely related to the cultured low GC content ultra-small marine *Actinobacteria*, namely Candidatus Actinomarina minuta (KC811150, Ghai et al., 2013), and to uncultured bacteria from surface seawater or the ultra-oligotrophic waters of the South Pacific gyre (JN985949.1).

### Surface Analysis of the Basaltic Glass

Estimates of the initial Fe redox state of the basaltic glass, based on Fe K-edge XANES analysis, confirmed that after synthesis, the oxidized basaltic glass BO2 contained around 97% Fe(III) whereas the reduced BH2 contained around 82% Fe(II) (see Supplementary Table S2).

**FIGURE 4:**
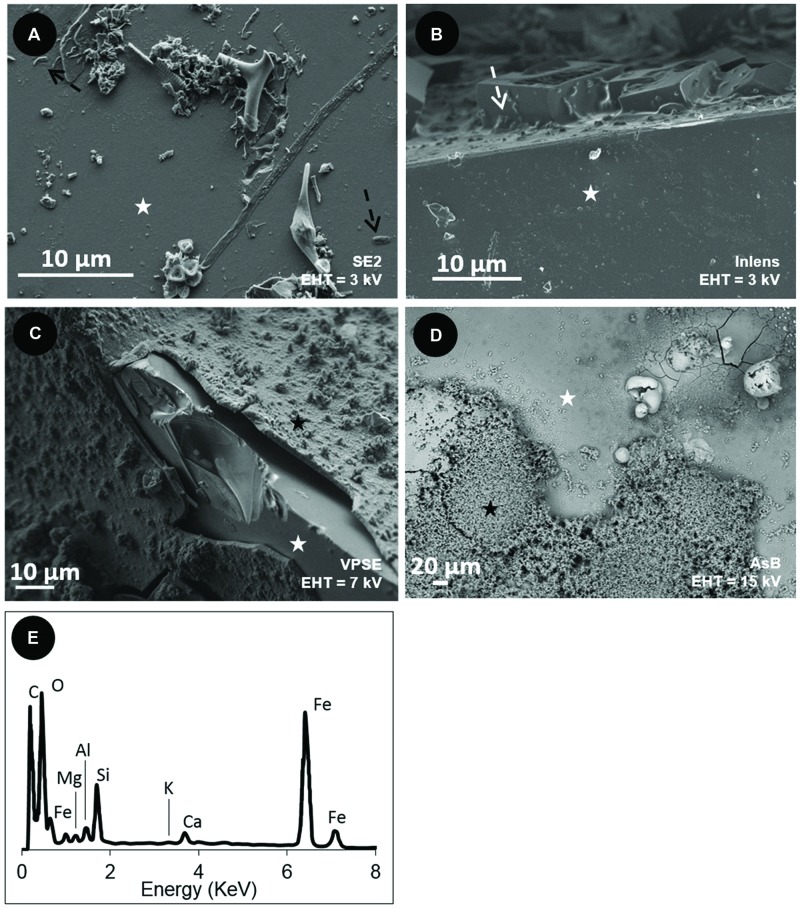
**Scanning Electron Microscopy (SEM) images of the surface of the basaltic glass chips incubated *in situ* in the abyssal plain **(A,C)** and *in vitro***(B,D)**. (A)** oxidized basaltic glass incubated *in situ* (BO2; seen from above); **(B)** reduced basaltic glass abiotically incubated *in vitro* at 4°C and serving as control (side view); **(C)** reduced basaltic glass incubated *in situ* (BH2; side view); **(D)** reduced basaltic glass incubated *in vitro* with *Mariprofundus ferrooxydans* DIS-1 (seen from top). White stars = basaltic glass surface; black stars = iron oxides; black dotted arrows = cell-like structures; white dotted arrows = salt crystals. **(E)** EDX spectrum collected on the weathering rind at the surface of the reduced basaltic glass (BH2) incubated *in situ* and shown in **(C)**. Accelerating voltage (EHT) and detection modes (SE2, VPSE, Inlens = secondary electrons; AsB = backscattered electrons) used for each image are indicated.

After incubation, the surfaces of the basaltic glass chips from the different experiments were observed using SEM and synchrotron X-ray fluorescence imaging. SEM observations revealed different features that depended upon the iron speciation in the initial basaltic glass (**Figure 4**). The BO2 samples from the *in situ* experiment did not show any traces of weathering (e.g., surface irregularities, dissolution pits) or secondary mineralization (**Figure 4A**), but cell-like structures and relics of diatoms and flagellates were observed that likely sank from the water column. Similarly, the *in vitro* abiotic control, performed using BH2, did not show any traces of cells or alteration features and only NaCl crystals formed during sample drying were noticeable (**Figure 4B**).

In contrast, the surface of BH2 incubated *in situ* had a quite uniformly thick (up to 150 μm) rust-colored rind that was evenly distributed on the surface compared to the *in vitro* incubated sample that had a thin rind sporadically distributed on the surface (**Figures 4C,D**, respectively). The BH2 rind was mainly enriched in iron and oxygen as indicated by EDX spectrum (**Figure 4E**) with less abundant Si, Al, K, Mn hence suggesting it was composed of iron oxides and silicates, likely clays, both being mineral phases composing palagonite that derives from glass alteration.

**FIGURE 5:**
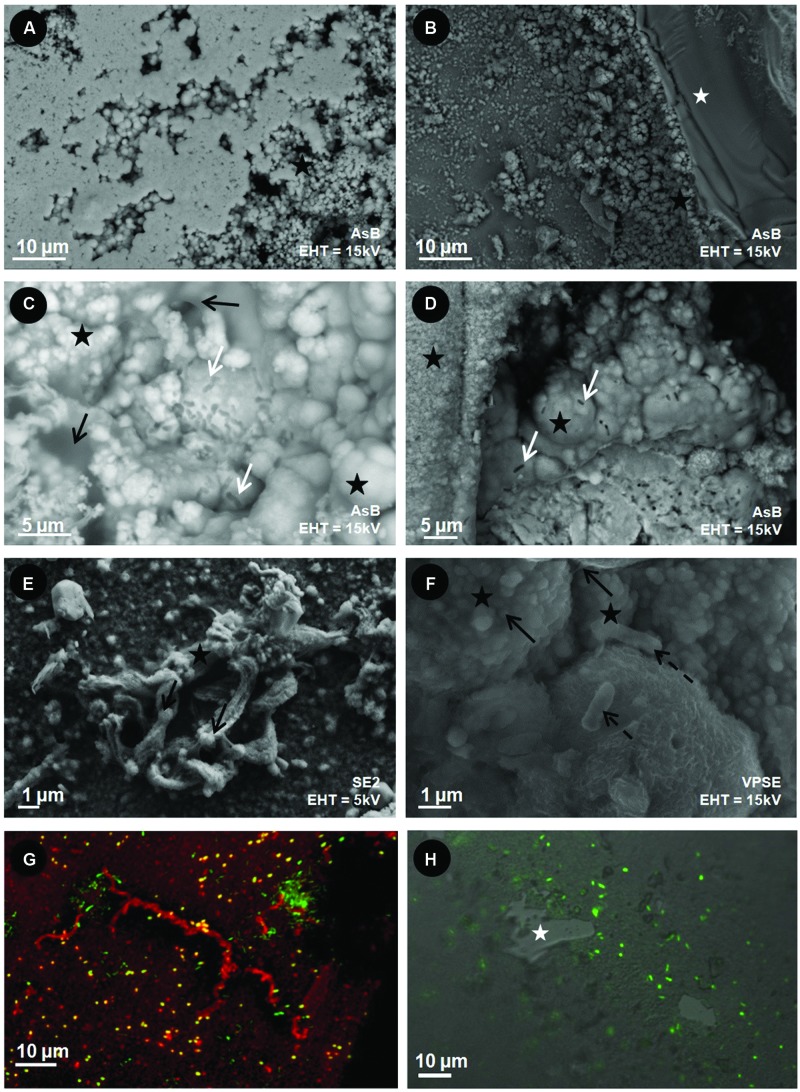
**Scanning Electron Microscopy **(A–F)** and CLSM **(G,H)** images of the surface of the reduced basaltic glass (BH2) incubated *in vitro* with *M. ferrooxydans* DIS-1 (left) and *in situ* in the abyssal plain (right)**. White stars = basaltic glass surface; black stars = iron oxides; black dotted arrows = cell-like structures; white arrow = cell imprints; black arrows = organic matter. **(G)** Composite CLSM image obtained with a sequential excitation at 488 and 555 nm and fluorescence emission collected between 300–550 and 578–800 nm, respectively; Green = SYTO^®^13 (DNA dye); Red = rhodamine-conjugated *Ricinus communis* agglutinin I (aliphatic chains’ marker); **(H)** CLSM image obtained with a excitation at a wavelength of 488 nm, by collecting the emitted fluorescence between 300 and 500 nm surperimposed on an image obtained in Differential Interferential Contrast; Green = SYTO^®^9 (DNA marker). Accelerating voltage (EHT) and detection modes (SE2, V PSE, Inlens = secondary electrons; AsB = backscattered electrons) used for each image are indicated.

The fine texture of the alteration rind appeared similar in samples from the *in vitro* and *in situ* experiments, both presenting Fe-rich aggregated spherules covering the surface of the basalt (**Figures 5A,B**). These micron-sized spherules and the associated larger ball-like structures often displayed numerous cell imprints at their surface and were partly covered by a veil of organic matter (**Figures 5C,D**). Higher magnification revealed the stalks were associated with microspherules for the *in vitro* experiment (**Figure 5E**) while in the *in situ* experiment aggregates of microspherules were interspersed with rod-shaped microbial cells without stalks (**Figure 5F**).

Microbial cells were closely associated to the iron-rich rind as revealed by CLSM images of the SYTO^®^9-stained BH2 sample from the *in situ* experiment (in green on **Figure 5H**). The reduced basaltic glass sample incubated in the laboratory flow-through system for 2 weeks with *M. ferrooxydans* exhibited a relatively high cell density and numerous Fe-bearing twisted stalks produced by the bacteria (**Figure 5G**). The laboratory incubation confirmed that microbial growth occurred presumably from Fe(II) supplied from the reduced basaltic glass, since it was the only iron source.

**FIGURE 6:**
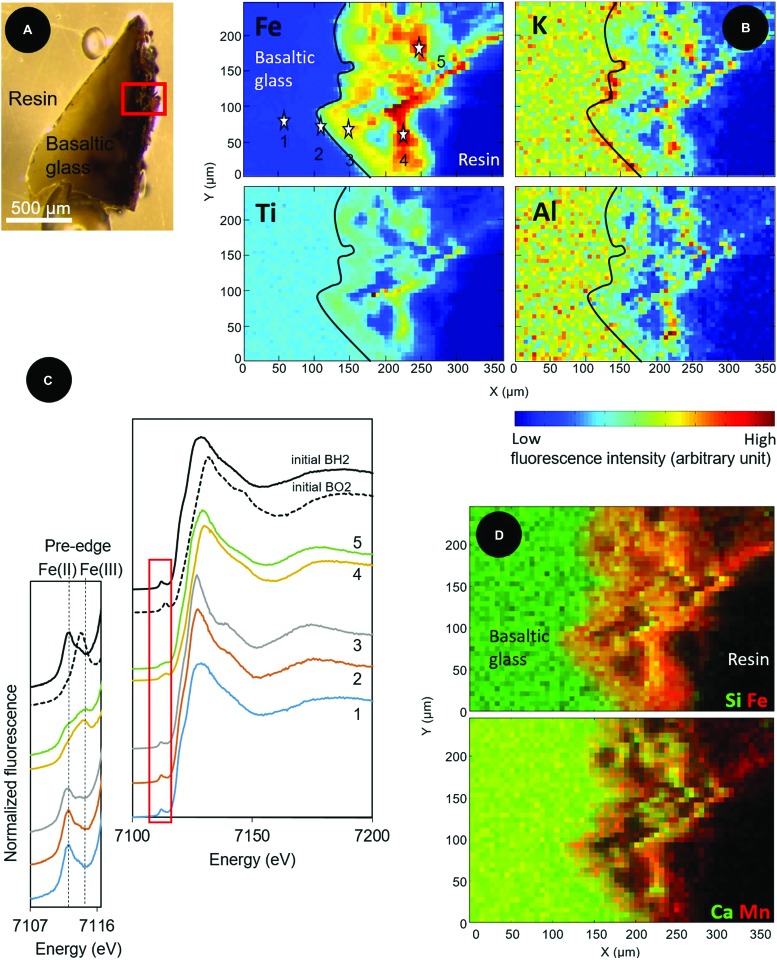
**Optical and X-ray fluorescence microprobe images along with XANES spectra at the Fe K-edge collected on a transversal section of a basaltic glass fragment incubated *in situ* in the abyssal plain. (A)** Optical image in reflected light of the resin-embedded BH2 fragment; **(B)** X-ray fluorescence maps of Fe, Ti, Al, and K collected at 8 keV in the area indicated by a red square in **(A)**. The black line delineates the alteration front; **(C)** Associated Fe K-edge XANES spectra collected from 4 μm × 4 μm spots at the numbered stars’ locations indicated on the Fe map in **(B)** and on unreacted reduced and oxidized basaltic glasses (BH2 and BO2) serving as references to demonstrate a change in oxidation state from Fe(II) in the basaltic glass to Fe(III) in the alteration rind.; **(D)** Red/Green maps of the distribution of Si (green) and Fe (red); and Ca (green) and Mn (red), showing the spatial colocation of Si and Ca, inversely correlated to the location of Fe and Mn.

Similarly, the elemental distributions produced by the X-ray fluorescence microprobe on a transversal section of a BH2 fragment (**Figure 6A**) showed an enrichment in iron in the 150-μm thick iron-oxide rind (**Figures 6B,D**). To a lesser extent, Ti was also enriched in the alteration rind, compared to the intact basaltic glass while the presence of Al is quite comparable between the two phases and K seems slightly enriched at the interface (**Figure 6B**). The rind appeared chemically heterogeneous with a second phase depicted by the presence of Al, K, Ca, and Si that are colocalized and inversely correlated with Fe and Mn distributions (**Figure 6D**). The SEM-EDX results indicated a partially crystallized palagonite which is constituted by a mix of Ca-, and Si-bearing clays and zeolites along with Fe-, Mn-bearing oxides and results from low-temperature alteration of basaltic glass (Knowles et al., 2012).

XANES spectra at the K-Fe edge were acquired in the unaltered core of the basaltic glass fragment (point 1 on **Figure 6B**), at the interface between the basalt and the alteration rind, likely constituting the alteration front (point 2) and at different locations in the iron-enriched rind (points 3, 4, and 5). The pre-edge features of each spectra showed that the proportion of Fe(III) progressively increased from the unaltered basalt (where it initially reached 18%) to the external part of the rind (**Figure 6C**). Fitting of the most outer spectrum (spectrum 5) using linear combinations of pure components yielded a closest match with the following combinations: 49.9% BH2, 23.3% hypersthene (pyroxene with a 2+ valence state for iron), 21.5% hematite, and 5.5% biotite, a clay mineral (Supplementary Figure S3).

**FIGURE 7:**
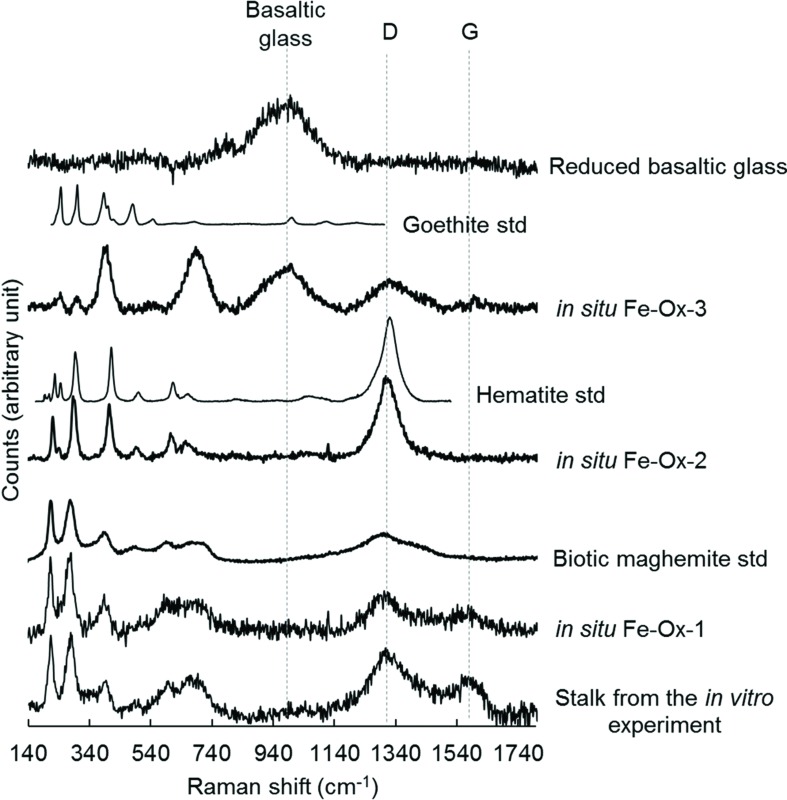
**Raman spectra collected at 514 nm excitation on iron oxides found in the alteration rind at the surface of the reduced basaltic glass incubated *in situ* in the abyssal plain and *in vitro* with *M. ferrooxydans* DIS-1, all compared to reference spectra of iron oxides (goethite, hematite, biogenic maghemite)**.

Raman spectroscopy was used to characterize the nature and mineralogy of the Fe-rich rind occurring at the surface of the chips of basaltic glass incubated both *in situ* and *in vitro*. As shown in **Figure 7**, the Raman spectrum of the basaltic glass typically exhibited a broad band around 975 cm^-1^. Depending on the thickness of the alteration rind, this band can be visible in some spectra showing also iron oxides’ characteristic bands. The Raman spectra collected in these Fe-rich areas also showed broad bands at 1320 and 1590 cm^-1^, identified as the *D* and *G* bands that are characteristic of poorly ordered carbonaceous material (Spötl et al., 1998) and interpreted as degraded organic matter according to previous studies (Maquelin et al., 2002; Callac et al., 2013). The comparison with spectra from reference compounds indicated the different iron (oxyhydr)oxides were either goethite [α FeO(OH)] for the Fe-Ox-3 point shown in **Figure 7** or biotic maghemite (γ Fe_2_O_3_) for the Fe-Ox-1 point. In other spectra, as the Fe-Ox-2 spectrum displayed in **Figure 7**, hematite (α Fe_2_O_3_) can be evidenced without any organic matter bands. Raman spectra of the Fe-bearing twisted stalks obtained during the *in vitro* experiment with *M. ferrooxydans* DIS-1 revealed a very close similarity with the Fe-Ox-1 spectrum obtained on the *in situ* incubated sample, both resembling the biotic maghemite standard. All these (oxyhydro)oxides are poorly crystallized, as revealed by their Raman band widths.

## Discussion

The mean temperature of 2.92°C recorded by the temperature probe for the entire duration of the incubation experiment (11 months) confirmed that the *in situ* incubation experiment was performed in a non-hydrothermally influenced environment, as expected for bottom seawater in an abyssal plain. Concordantly, the dominance of the SAR11 clade and the co-occurrence of other clones indicative of marine heterotrophs in the water sample were consistent with an ambient seawater community. Similarly, the important contribution in the sediment sample diversity of OTUs closely related to sequences from deep sea sediments and polymetallic nodules was also in agreement with such a sedimentary abyssal environment.

Conversely, the communities of bacteria on the *in situ* incubated basaltic glasses (both BO2 and BH2) were distinct from surrounding seawater. Indeed the SAR 11 clade that dominated in the water sample was absent in both basalts’ surface communities. Instead, the sequences retrieved from the incubated basalts were related to those extracted from deep sea sediment or to marine symbiotic bacteria (of either wood boring bivalves or gastropods and chitons). Symbionts of *O. mucoflorix* were particularly represented, with relatives belonging to the Alphaproteobacteria, Gammaproteobacteria, and Epsilonproteobacteria classes that encompass sulfide oxidizers. The presence of Alphaproteobacteria (other than those from the SAR11 clade), Gammaproteobacteria, Epsilonproteobacteria (sulfide-oxidizers) along with *Planctomycetes* and *Bacteroidetes* is fully consistent with previous studies documenting microbial communities at the surface of relatively young outcropping basalts (Lysnes et al., 2004; Santelli et al., 2008; Mason et al., 2009; Sudek et al., 2009).

The occurrence and abundance of Zetaproteobacteria is, however, less common but seems also related to the presence of basalt. Indeed, numerous sequences, besides the Zetaproteobacteria sequences, retrieved from BH2 were similar to environmental sequences collected in hydrothermal environments and seamounts or in basalts from various locations. Orcutt et al. (2011) described both microbial communities at the surface of different colonized solids (including basalt) incubated *in situ* during 4 years at depths of 210 and 280 m below seafloor (mbsf) in a 3.5 Ma drilled basaltic crust, and the alteration phases present at the surface of the colonizable solids. In Orcutt’s study, the surfaces of the incubated solids were mostly colonized by *Firmicutes*, the candidate class OP18 and Gammaproteobacteria whatever the type of minerals, and reflected an anaerobic community. Although not inventoried in the microbial diversity, the surfaces of basalt chips in those incubations showed evidence for helical filaments composed of iron oxides, resembling those produced by iron-oxidizing bacteria such as Zetaproteobacteria, suggesting the presence of iron-oxidizing organisms during the course of the colonization experiment, perhaps during an earlier, more aerobic phase of the experiment. In the present study, the samples were incubated hundreds of kilometers away from the nearest known hydrothermal field or from any fresh basalt outcroppings (associated with ridge processes). This suggests that there are either unrecognized resources that support the growth of these basalt-adapted microbes on the seafloor, or that they are part of a dormant, rare biosphere that seed the deep ocean, and bloom when they encounter environmental conditions that support their growth.

The most remarkable difference between the reduced and oxidized basaltic glasses was the presence and high relative abundance of Zetaproteobacteria on the reduced glass, but their absence on the oxidized one along with the concomitant presence of an alteration rind. A recent meta-analysis of Zetaproteobacteria distribution found they were only present in high Fe(II) marine environments where they are assumed to be playing an important role in iron-oxidation under microaerophilic conditions (Scott et al., 2015). Their presence in this deep (3200 mbsl), cold and oxygenated environment with a low level of Fe(II) because far from any hydrothermal activity suggests they may have been recruited from cold sediments where a biogenic iron cycle is present. Although Fe-oxidizing Zetaproteobacteria were first described at low temperature, Fe-rich diffuse vents (Moyer et al., 1995; Davis et al., 2009; Rassa et al., 2009; Forget et al., 2010) more recent studies have documented their presence in diverse habitats including non-hydrothermal environments (McAllister et al., 2011; McBeth et al., 2011; Rubin-Blum et al., 2014). To account for the presence of Zetaproteobacteria in continental margin sediments with no hydrothermal activity, Rubin-Blum et al. (2014) proposed that the particulate Fe from the water column is likely reduced in microenvironments and thus become bioavailable for FeOB inhabiting the sediment. Nonetheless, in our study, Zetaproteobacteria were only retrieved from the reduced basaltic glass samples and were not detected on the oxidized basaltic glass, in the surrounding seawater or in the sediment. Rarefaction curves showed, however, that the sampling effort was likely insufficient, particularly for the sediment, to fully embrace the entire diversity. We thus propose that Zetaproteobacteria colonizing the BH2 sample could come from the sediment where Fe(II) concentrations are either too low, or too localized, to allow these organisms to sufficiently develop to be detected by classical Sanger sequencing. Interestingly, an enrichment experiment carried out in a near-shore environment of the Atlantic using mild steel also resulted in abundant growth of Zetaproteobacteria (McBeth et al., 2011). Similar to the results reported here, while the relative abundance of Zetaproteobacteria on the steel surface was high, the diversity was very low, being represented by only 1 or 2 OTUs. We suggest that they were recruited from a low abundance population of the surrounding seawater where a modest biologically driven iron cycle occurs as in the deep-sea sediments where our experiment was conducted.

We also propose the presence of Fe(II) in BH2 to be key in the specific colonization process, hence supporting growth of these FeOB. The 4–10 wt% Fe in the reduced basaltic glass, as determined by semi-quantitative EDX analyses, represented the most abundant source of Fe(II) in the *in situ* experiment. If Fe(II) would have come from another source (either as dissolved or particulate Fe(II) or as Fe(III) precipitates from seawater), the oxidized basalt would also have shown the presence of Zetaproteobacteria in its community along with an alteration rind as the one observed for BH2. The hypothesis that the source of iron was the structural Fe(II) of the reduced basaltic glass is also supported by the results obtained in the *in vitro* experiment where the Fe(II) from the reduced basalt was used by Zetaproteobacteria as the primary energy source for their growth. Regarding the relative abundance of Zetaproteobacteria in the *in situ* incubated samples, the enrichment in Fe(II) provided by the reduced basalt was sufficient to provoke a “bloom” of sediment-hosted Zetaproteobacteria.

After less than 1 year of incubation, the state of alteration in both the *in situ* and *in vitro* experiments is likely too early to show a strongly irregular basalt surface or noticeable hemispherical peats that could results from cells-basalts interactions as observed in numerous studies on old basalts (mostly older than 1 Ma; Wilke et al., 2005; Thorseth et al., 1992, 1995, 2001, 2003; Furnes and Staudigel, 1999; Staudigel et al., 2008). That is why we will further designate a significant alteration by the presence of an alteration rind of iron oxyhydroxides, since the secondary mineral phases resulting of the basalt alteration are mostly composed of iron oxyhydroxides and clays (Knowles et al., 2012).

The presence and relative abundance of Zetaproteobacteria in the BH2 sample correlated with the presence of a thick rind of alteration composed of poorly crystallized iron oxyhydroxides along with Si-Al-Ca-K bearing phases as evidenced by SEM-EDX and synchrotron X-ray fluorescence measurements. This alteration rind attests for the mobilization (dissolution and reprecipitation) of the basalt-forming elements into secondary phases. It shows striking similarities with palagonite, a byproduct of the low temperature alteration of basaltic glass, composed of amorphous or poorly crystallized iron (oxyhydr)oxides together with clay minerals. Nonetheless their biological or abiotic origin is often tricky to assess (Knowles et al., 2012). As confirmed by the CTD profile, the seawater at this depth is oxygenated and deep seawater pH in the North Atlantic is close to circumneutral values. Abiotic iron oxidation occurs rapidly under these conditions and the BH2 alteration rind could then have been formed at least partly abiotically. Nonetheless, both the SEM observations of the rind-hosted iron-oxides showing them associated to microbial cells and the Raman spectra of the Fe-oxides very often associated to organic matter, suggest a biological origin for these Fe-precipitates (**Figures 5** and **7**). Additionally, the abundant presence of microaerophilic Zetaproteobacteria in the reduced basaltic glass sample suggests that a low-oxygen micro-environment may have formed in the recesses of the stacked basaltic glass fragments likely due to weak circulation of oxygenated seawater. It is also possible that a biofilm aggregating the basalt chips or the alteration rind itself played the role of diffusion barrier for oxygen. This weakly oxygenated micro-environment could have permitted Fe(II) to be available for oxidation by Zetaproteobacteria. Coherently, during the abiotic *in vitro* incubation of the reduced basaltic glass at 4°C for a period of time that is comparable to the one of the *in situ* experiment, no alteration rind was observed (**Figure 4**). This suggest that no basalt dissolution occurred abiotically at seafloor temperatures over 1 year and that Zetaproteobacteria likely play a dynamical role in extracting Fe(II) from the basalt and hence in altering the glass.

The textures of the iron oxides observed at the surface of *in situ* incubated BH2 did not show the Fe-twisted stalks typifying *M. ferrooxydans* (Kato et al., 2009; Rassa et al., 2009; McBeth et al., 2011; Fleming et al., 2013; Rubin-Blum et al., 2014). However, several FeOB including *Sideroxydans* sp., and *Gallionella capsiferriformans* are known to produce amorphous iron oxides (Emerson and Moyer, 1997, 2002; Weiss et al., 2007); furthermore, a non-stalk-forming member of the Zetaproteobacteria that is an iron oxidizer was recently isolated from an Fe(II)-rich, diffuse hydrothermal vent site at TAG (Emerson, unpublished). The stalks produced by FeOB are coated by extracellular polymeric substances (EPS). The organic matter is presumed to be excreted by cells in order to bind and precipitate the Fe(III) produced by Fe(II) oxidation away from the cell to avoid encrustation (Chan et al., 2011). The same process has been suggested for FeOB that do not produce stalks (Miot et al., 2009). The association of organic matter with the poorly crystallized iron oxides evidenced by Raman spectroscopy in BH2 sample could be related to such a process. Moreover, the Raman spectra obtained on the stalk-organized iron oxides from the *in vitro* experiment with *M. ferrooxydans* DIS-1 were very similar to those obtained on the non-organized iron oxides from the *in situ* experiment (**Figure 7**). According to the phylogenetic distance with the cultured strains (**Figure 3**), the 16S rRNA gene sequence of Zetaproteobacteria retrieved in the abyssal plain could belong to a new genus able to oxidize Fe(II) and to embed in organic matter the produced Fe(III) to avoid cell incrustation but without creating twisted stalks. Another hypothesis derived from the similarity at low magnification of the alteration features in between the *in vitro* and the *in situ* incubated reduced basaltic glass (**Figures 5A–D**) is that the stalks have been formed, and then some processes led to their disappearance. They could have either been dissolved, re-crystallized, silicified (Li et al., 2013) or reduced by iron-reducing bacteria (Lee et al., 2013).

**FIGURE 8:**
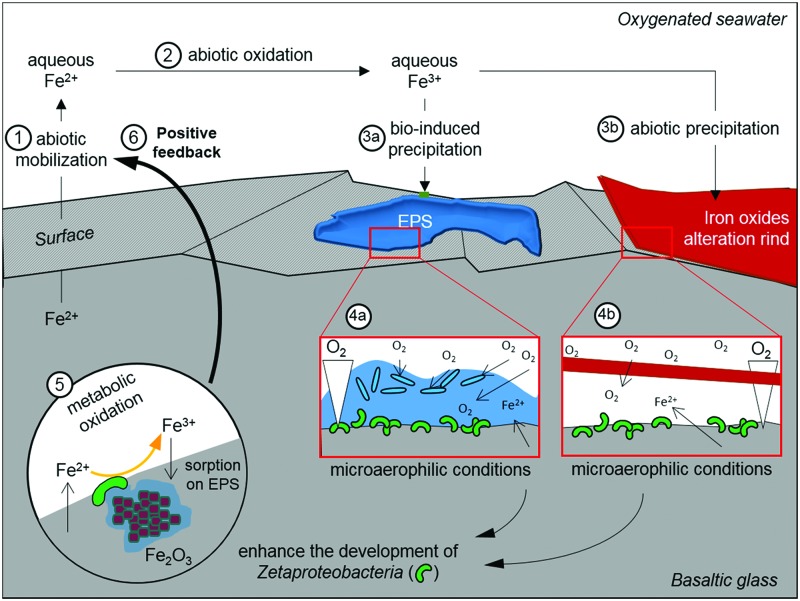
**Schematic representation of the postulated alteration process that has affected the reduced basaltic glass during the *in situ* incubation experiment in the abyssal plain**. EPS stands for extracellular polymeric substances.

Overall, based on the results of both the *in situ* and *in vitro* experiments, we propose that the important rind of alteration observed solely at surface of the reduced basaltic glass BH2 derived from the development of iron-oxidizing Zetaproteobacteria. The basalt weathering has then been enhanced by their metabolic activity through the mechanisms depicted in **Figure 8**: the dissolution of the basaltic glass first occurred abiotically, hence releasing structural Fe(II) (1 on **Figure 8**); the dissolved Fe(II) was then quickly abiotically oxidized due to the oxygenated environment (2). The subsequent precipitation of insoluble Fe(III) could have been either bio-influenced (Decho, 2010) by the EPS produced by diverse microorganisms (3a) or abiotic, but in all cases would lead to the formation of an iron oxide alteration rind (3b). Microaerophilic conditions were then created either by (4a) the biofilm (the oxygen could have been consumed by diverse aerophilic microorganisms on the top of the biofilm hence leading to a decreasing gradient of oxygen concentration up to the basalt surface (De Beer et al., 1994; Xu et al., 1998) or (4b) the thick alteration rind that formed a barrier of diffusion for the oxygen (Schott and Berner, 1983, 1985; Chou and Wollast, 1984). The microaerophilic conditions allowed the development of Zetaproteobacteria (5) that metabolically converted the structural Fe(II) into Fe(III). To avoid cell encrustation, Fe(III) was then quickly adsorbed on EPS (Banfield et al., 2000; Chan et al., 2009). EPS promoted precipitation through the presence of diverse functional groups negatively charged (e.g., hydroxyl, carboxyl) and acting as nucleation sites, but also inhibiting the growth of iron oxides, hence leading to small particles (Vollrath et al., 2013) as those observed coated by EPS (**Figure 5F**). Aggregation of these particles contributed to the alteration features observed at low magnification (**Figure 5B**). The removal of Fe(III) induced a positive feedback on Fe(II) dissolution by the modification of the system equilibrium, as observed with the acidophilic FeOB *Acidithiobacilus ferrooxydans* on basaltic glass by Navarrete et al. (2013).

## Conclusion

In this study, we reported the abundant presence of Zetaproteobacteria on a reduced basaltic glass incubated a sedimentary abyssal plain and we demonstrated by both *in situ* and *in vitro* experiments that the structural Fe(II) of the basaltic glass can be used as the sole energy source for the development of FeOB in a non-hydrothermal environment. The outcropping pillows of fresh basalts at seafloor hence represent a potentially important habitat and energy source to sustain the development of FeOB and particularly Zetaproteobacteria. In terms of volume and contribution of these FeOB to the iron cycle and rock weathering at the interface between the oceanic lithosphere and the deep ocean this process could be very important in Earth history.

## Conflict of Interest Statement

The authors declare that the research was conducted in the absence of any commercial or financial relationships that could be construed as a potential conflict of interest.
