# Supplementary material for: Structural Iron (II) of Basaltic Glass as an Energy Source for Zetaproteobacteria in an Abyssal Plain Environment, Off the Mid Atlantic Ridge
Source: Front Microbiol. 2016 Jan 21;6:1518. doi: 10.3389/fmicb.2015.01518 (PMC4720738; doi:10.3389/fmicb.2015.01518)
Supplement: Supplementary file 1 [file Presentation_1.PDF]

Supplementary Material

Structural iron (II) of basaltic glass as an energy source for Zetaproteobacteria in an abyssal plain environment, off the Mid Atlantic Ridge

Pauline Henri[1]*, Céline Rommevaux-Jestin[1], Françoise Lesongeur[2], Adam Mumford[3], David Emerson[3], Anne Godfroy[2] and Bénédicte Ménez1

[1] *Institut de Physique du Globe de Paris, Sorbonne Paris Cité, Univ Paris Diderot, CNRS, Paris, France*

[2] Laboratoire de Microbiologie des Environnements Extrêmes, Ifremer, CNRS/UMR 6197, Plouzané, France

[3] Bigelow Laboratory for Ocean Sciences, East Boothbay, ME, USA

*Correspondence:

Pauline Henri
Institut de Physique du Globe de Paris, IPGP, UMR 7154
Laboratoire Géobiosphère Actuelle et Primitive
1 rue Jussieu
75238 Paris Cedex 05, France
henri@ipgp.fr

1. Supplementary Data

Synthesis of the basaltic glasses

The initial oxides' or carbonates' powders were first dried at 150°C for at least 24 hours in an oven before being mixed and homogenized in an agate mortar and then placed in a platinum crucible. Basalt glasses have been synthesized under either reducing (H_2) or oxidizing atmosphere (O_2) in a vertical furnace with automated quenching system. The temperature was brought from 40 to 600°C over two hours and stabilized at this last value for forty-five minutes to allow decarbonation. The temperature was then brought to 1500°C over three hours and stabilized at this last value for fifty minutes before immediate quenching. After synthesis, glasses were crushed into 1 to 4 mm sized fragments in an agate mortar. Surfaces of the obtained fragments were rough and not planar.

454-pyrosequencing full protocol

For the 454-pyrosequencing, we amplified the V2-V3 region of the 16S rRNA coding genes with 27F (5'-AGA GTT TGA TCC TGG CTC AG-3') as the forward primer and 533R (5'-TTA CCG CGG CTG CTG GCA C-3') as the reverse one. Each couple of primers used for each samples had a multiplex identifier (MIDs) allowing identifying the origin of the amplicons after pooling the PCR products. In order to decrease the bias associated with the use of MIDs, the same DNA sample was amplified with two different ones (*i.e.* MID _1: 5'-ACG CTC GAC A-3'; MID _2: 5'-ACG ACT ACA G-3'). The reaction medium was prepared as follows: 19 µl of DNA-free water, 0.75 µl of $MgCl_2$ (at 50 µM), 2.5 µl of Taq Buffer X10, 0.5 µl of dNTP mixture (at 10 mM each), 0.75 µl of primer (10 µM each) and 0.15 µl of platinium Taq DNA polymerase (Invitrogen^{TM}). One to 2 µl of DNA was added to the final reaction mix for each sample. Amplifications were carried out by using the following conditions: first, denaturation during 3 min at 94°C and then 30 cycles at 94°C for 30 s, 50°C for 45 s and 72°C for 1 min 30, with a final extension at 72°C for 10 min. To counterbalance PCR bias, we performed 10 PCRs per MID. We pooled together the 10 PCR products before purification by the QIAquick® PCR Purification Kit (Qiagen). DNA concentrations of each sample were controlled through DNA absorbance at 260 nm in a UV-visible spectrophotometer (NanoDrop 2000, Thermoscientific) in order to ensure the equimolarity of each sample in the final mix. A total amount of 4 µg DNA was sent to Beckman Coulter genomics (Danvers, Massachusetts, USA) for sequencing using the Roche GS FLX platform (454 Life Sciences, Branford, CT) with the Titanium LIB-A kit for bi-directional amplicons sequencing. Demultiplexing and contigs' assembling have also been carried out by Beckman Coulter genomics.

Sanger sequencing full protocol

Sequencing of the 16S rRNA coding genes of the DNA extracted from the reduced and oxidized incubated basaltic glasses (BH2 and BO2, respectively), the abyssal plain sediment and the seawater samples were performed by conventional Sanger techniques, by using universal primers: U1492R (5'-GGC TAC CTT GTT ACG ACT T-3') as reverse primer and E8F (5'-AGA GTT TGA TCC TGG CTC AG-3') or 27F (5'-AGA GTT TGA TCC TGG CTC AG-3') as forward primers. The reaction medium was prepared as follows: 36.36 µl of DNA-free water, 10 µl of Go Taq Buffer X5 containing $MgCl_2$, (Invitrogen^{TM}), 1 µl of dNTP mixture (at 10 mM each), 0.2 µl of primer (100 µM each) and 0.24 µl of GoTaq DNA polymerase (Invitrogen^{TM}). One to 2 µl of DNA was added to the final reaction mix for each sample. Amplifications were carried out by using the following conditions: first, denaturation during 3 min at 94°C and then 30 cycles at 94°C for 1 min, 50°C for 1 min 30 and 72°C for 2 min, with a final extension at 72°C for 6 min. PCR products were visualized using 1% agarose gel electrophoresis. Clone libraries were constructed using the TOPO® XL PCR Cloning Kit, with the One Shot® TOP10 Chemically Competent *Escherichia coli* kit (Invitrogen^{TM}) according to the manufacturer instructions. Plasmid extraction, purification and sequencing were carried out by GATC Biotech (Germany).

XANES: Samples preparation and analysis

Resin-embedded samples and references were mounted on a copper slide using glue or silver lacquer and placed perpendicular to the beam. A Si(Li) detector collected the emitted fluorescence at 90° with respect to the incident beam. Fe K-edge XANES spectra were collected between 7107.1 and 7118 eV with energy increment of 0.1 eV, between 7118.3 and 7155 eV with energy increment of 0.3 eV, between 7155.5 and 7222 with energy increment of 0.5 eV and between 7223 and 7300 with energy increment of 1 eV, all with a counting time of 1s per points. To improve the signal-to-noise ratio and rule out beam damage or possible photooxidation by the beam, two spectra were collected at each point. Data reduction, including duplicated spectra averaging, background subtraction and normalization, was conducted using the ATHENA 0.8.056 software (Ravel and Newville 2005).

2. Supplementary Figures and Tables

2.1. Supplementary Figures

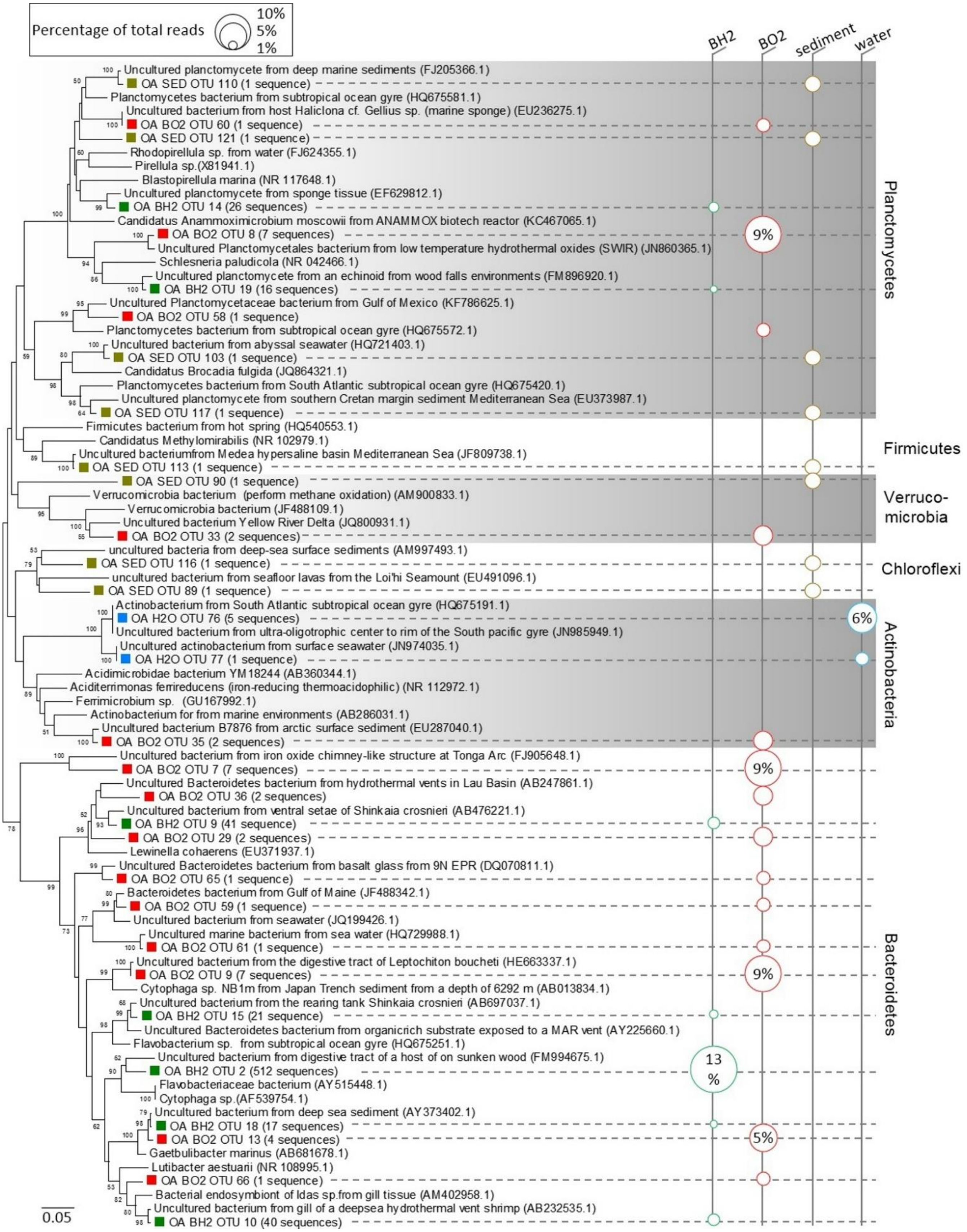

Supplementary Figure S1

Supplementary Figure S1 (continuation): Phylogenetic relationships among the non-proteobacterial 16S rRNA gene sequences of representative OTUs retrieved from the oxidized (BO2) and the reduced (BH2) basaltic glass incubated in the abyssal plain along with the seawater (H2O) and the sediment (SED), with the same legend as in Figure 3.

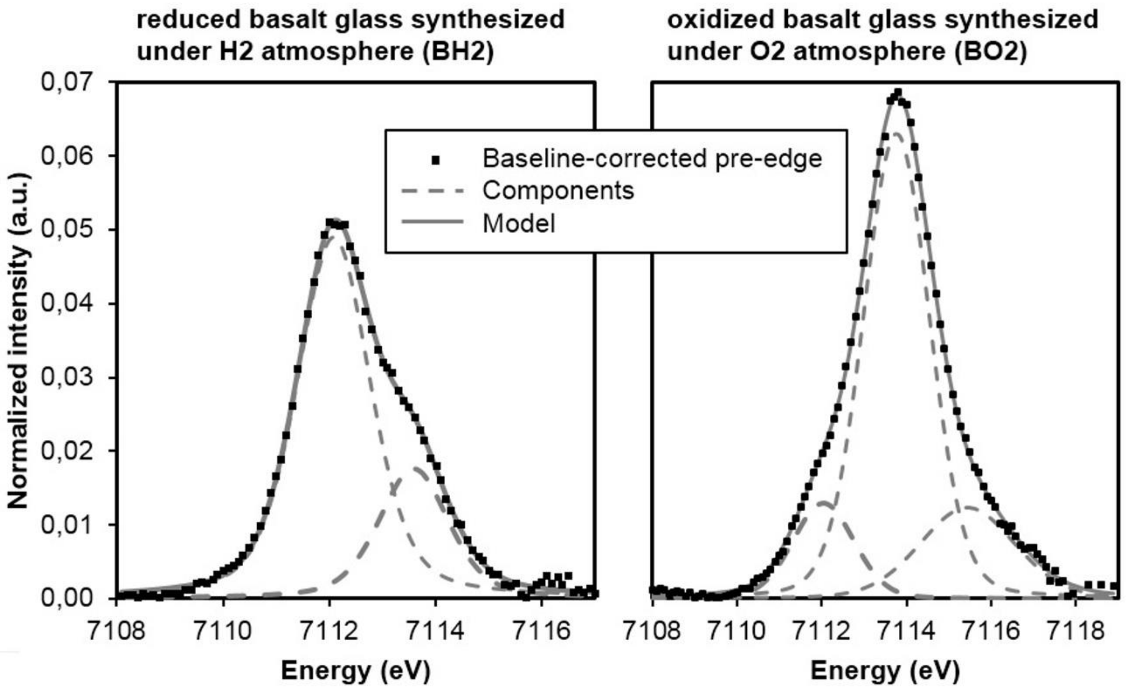

$$\frac{Fe(III)}{\sum Fe * 100} = \frac{\left[-0.028 + \left(0.000784 + 0.00052 * (7112 - C)\right)^{0.5}\right]}{-0.00026}$$

Supplementary Figure S2: Determination of the basaltic glass redox state by XANES measurement at the Fe K-edge (pre-edge fitting). In the used formula, *C* is the Centroïd and corresponds to the integrated intensity weighted-average of the components' positions with 7112 ≤ *C* ≤ 7113.5 eV (Wilke et al. 2005).

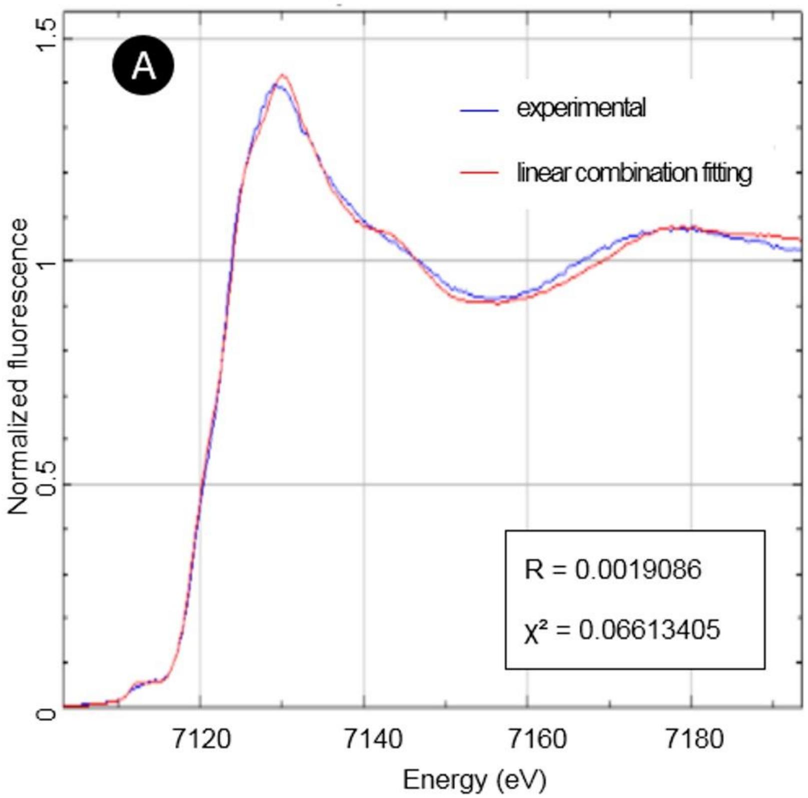

B

Standards	Contribution to the linear combination fitting (%)
initial BH2 glass	49.9
Hypersthene	23.3
Hematite	21.5
Biotite	5.5
Biotic maghemite	0
Lepidocrocite	0
Goethite	0
Ferrihydrite	0

Supplementary Figure S3: Linear combination fitting of the XANES spectrum collected at the Fe K-edge on the transversal section of a basaltic glass fragment incubated *in situ* in the abyssal plain (A). This spectrum corresponds to the location of the spot numbered 5 and indicated by a star on the Fe map shown in Figure 6-B. The respective contributions to the linear combination fitting of the different standard spectra used in the fitting are indicated in percentage in (B).

2.2. Supplementary Tables

Supplementary Table S1: Standards of silicates, oxides and iron carbonate used and analyzed during the XANES experiment at the Fe K-edge.

	MINERAL	FORMULA	TYPE
OXIDES	Hematite	α-$Fe(III)_2O_3$	powder and pellet
	Goethite	α-$Fe(III)O(OH)$	powder
	Lepidocrocite	γ-$Fe(III)O(OH)$	powder and pellet
	Ferrihydrite	$Fe(III)_5O_3(OH)_9$	powder
	Biotic maghemite	γ- $Fe(III)_2O_3$	powder
	Hercynite	$Fe(II)Al_2O_4$	fragment
SILICATES	Biotite	$K(Mg, Fe(II))_3AlSi_3O_{10}(OH)_{1.75}$	fragment
	Hypersthene	$(Mg, Fe(II))SiO_6$	fragment
	Actinolite	$Ca_2(Mg, Fe(II))Si_8O_{22}(OH)_2$	fragment
	Enstatite	$Mg_2Si_2O_6$	fragment
IRON CARBONATE	Siderite	$Fe(II)CO_3$	fragment

Supplementary Table S2: Results of the fitting procedure for the reduced (BH2) and oxidized (BO2) synthetic basaltic glasses.

	PEAK TYPE	CENTRE	HEIGHT	AREA	TOTAL AREA	CENTROÏD	FE(III)/Σ FE*100
BH2	PseudoVoigt	7112.08	0.05	0.10	0.14	7112.46	18
	PseudoVoigt	7113.58	0.02	0.03			
BO2	PseudoVoigt	7113.77	0.06	0.13	0.16	7113.49	97
	PseudoVoigt	7112.04	0.01	0.03			
	PseudoVoigt	7115.45	0.01	0.04			

3. References

Ravel, B., and Newville, M. (2005). ATHENA and ARTEMIS: interactive graphical data analysis using IFEFFIT. *Physica Scripta* 2005, 1007.

Wilke, M., Partzsch, G. M., Bernhardt, R., and Lattard, D. (2005). Determination of the iron oxidation state in basaltic glasses using XANES at the K-edge. *Chemical Geology* 220, 143–161.
